# New rapid detection by using a constant temperature method for avian leukosis viruses

**DOI:** 10.3389/fmicb.2022.968559

**Published:** 2022-08-18

**Authors:** Xiuhong Wu, Fengsheng Chu, Luxuan Zhang, Sheng Chen, Liguo Gao, Hao Zhang, Haohua Huang, Jin Wang, Mengjun Chen, Zi Xie, Feng Chen, Xinheng Zhang, Qingmei Xie

**Affiliations:** ^1^Heyuan Branch, Guangdong Provincial Laboratory of Lingnan Modern Agricultural Science and Technology and Guangdong Provincial Key Lab of Agro-Animal Genomics and Molecular Breeding, College of Animal Science, South China Agricultural University, Guangzhou, China; ^2^Guangdong Engineering Research Center for Vector Vaccine of Animal Virus, Guangzhou, China; ^3^South China Collaborative Innovation Center for Poultry Disease Control and Product Safety, Guangzhou, China; ^4^School of Pharmaceutical Science, Sun Yat-sen University, Guangzhou, China

**Keywords:** avian leukosis viruses, p12, real-time reverse-transcription recombinase-aided amplification assay, detection by constant temperature, clinical diagnosis

## Abstract

The avian leukemia virus causes avian leukemia (AL), a severe immunosuppressive disease in chickens (ALV). Since the 1990s, the diversity of ALV subpopulations caused by ALV genome variation and recombination, and the complexity of the infection and transmission, with currently no effective commercial vaccine and therapeutic for ALV, has resulted in severe economic losses to the chicken business in various parts of the world. Therefore, as a key means of prevention and control, an effective, rapid, and accurate detection method is imperative. A new real-time reverse transcription recombinase-aided amplification (RT-RAA) assay for ALV with rapid, highly specific, low-cost, and simple operational characteristics have been developed in this study. Based on the amplification of 114 base pairs from the ALV P12 gene, real-time RT-RAA primers and a probe were designed for this study. The lowest detection line was 10 copies of ALV RNA molecules per response, which could be carried out at 39°C in as fastest as 5 min and completed in 30 min, with no cross-reactivity with Marek's disease virus, avian reticuloendothelial virus, Newcastle disease virus, infectious bronchitis virus, infectious bursal disease virus, infectious laryngotracheitis virus, and avian influenza virus. Furthermore, the kappa value of 0.91 (>0.81) was compared with reverse transcription–polymerase chain reaction (RT-PCR) for 44 clinical samples, and the coefficients of variation were within 5.18% of the repeated assays with three low-level concentration gradients. These results indicate that using a real-time RT-RAA assay to detect ALV could be a valuable method.

## Introduction

The avian leukosis viruses (ALVs) belong to the family type C *Retroviridae* (Walker et al., 2020), subfamily *Orthoretrovirinae*, and the genus *Alpharetrovirus*, which infect a variety of poultry and induce neoplastic diseases and severe immunosuppressive diseases (Payne et al., 1992, 1993). ALV transmission pathways can either be horizontal or vertical (Zhang et al., 2016), which is a key to our prevention and control of ALV. Based on their host range, interference with viral envelopes, and patterns of cross-neutralization, the ALV has been split into 11 subgroups, called A-K (Payne, 1992). Exogenous ALVs have subgroups A, B, C, D, J, and K, whereas endogenous ALVs only have subgroup E (Gao et al., 2014; Wang et al., 2019).

Most infected chickens show subclinical symptoms on farms, such as decreased egg production, immunosuppression, or growth retardation (Witter, 1997; Dai et al., 2021). The ALV subclinical infection is more severe than the clinically shown tumor death in terms of economic impact. At present, the prevalence of ALV in chicken flocks has been eliminated and controlled in most countries with developed chicken industry (Zhou et al., 2019), but ALV has caused great economic losses to the chicken industry of China and seriously harmed the healthy development of various types of chickens in China, for instance, white broilers, layers, yellow broilers, and inherent local breeds (Cheng et al., 2005; Li et al., 2018, 2019; Wen et al., 2018). It has grown into a serious disease that jeopardizes poultry industry development in China.

In China, exogenous ALV infection was dramatically reduced at major representative breeding farms when the Nationwide Eradication Program (NEP) was launched in 2008 (Wang et al., 2018, 2019). Despite this, there were still small outbreaks of exogenous wild ALV strains in native chicken breeds (Su et al., 2018) because of not carrying out strict implementation of effective prevention and control measures, the vast territory, and complex breeding situation of China.

Since the identification and isolation of ALV in China in 1999, researchers have continued to investigate the epidemiology of ALV. Before the administration implemented the NEP in 2008, ALV broke out in the poultry industry in several provinces, resulting in morbidity and mortality rates as high as 50% before the Chinese administration implemented the NEP in 2008 (Cheng et al., 2005, 2010; Sun and Cui, 2007), causing substantial economic losses to lay hens and the inherent local breeds. Although the ALV epidemic had decreased after 2008, the molecular epidemiological investigations of ALV between 2013 and 2018 revealed that gp85 and U3 genomic regions of local chicken strains had rapidly evolved to form a new unique branch, and genetic elimination in the 3′UTR area was a molecular feature of ALV in China (Cui et al., 2014; Dong et al., 2015; Ma et al., 2020). In terms of the host's genetic heritage and geographical location, analysis of homology over the entire ALV genome from 1988 to 2018 showed that ALV strains randomly changed the original strain HPRS-103 in different directions over time, and the variation of the *env* gene was the most significant (Wang et al., 2020). However, the U3 region of the ALV strain isolated from Pakistan was highly homologous to the ALV-J strain from the United States, and gp85 was more similar to the original strain HPRS103, which differed from the epidemic situation in China (Farooque et al., 2020). They discovered in Egypt that the ALV gp85 gene in a distinct group with a specific mutation was not the same as a strain obtained in 2005, but it was genetically identical to prototype virus HPRS-1003 (Yehia et al., 2020). Subsequently, at the end of 2018, white-feathered broiler chickens broke out with ALV again, and local flocks showed AL-like symptoms. What was more challenging was that new mutant ALVs were being bred with local chickens. There are no specific medications or vaccinations available to combat the ALV, which has a significant impact on the poultry industry's ability to thrive (Li et al., 2021). At present, our prevention of the ALV centers more on testing, and fortunately, testing can help us reduce outbreaks of the ALV. Any test method has two sides. As an example, virus isolation is the most reliable method, but it consumes too much labor force and resources. In this study, we established a new use of convenient, fast, and accurate detection methods to prevent and control the ALV.

RAA technology is a breakthrough thermostatic alternative method, combined with a portable GENCHEK fluorescence detector that can achieve rapid and accurate results within 5–20 mins at 39–42°C. It has been applied to detect harmful microorganisms, genetically modified organisms (GMO), disease pathogens, and other fields. For instance, researchers had applied the RAA detection in zoonosis of *Clonorchiasis sinensis* (Zhang et al., 2019), and harmful microorganisms of *Klebsiella pneumoniae* (Zhang et al., 2021). An RT-RAA detection method was also reported in the ASFV outbreak in China in 2018 (Bai et al., 2019; Zhang et al., 2021). Moreover, some studies on novel coronavirus 2019 recommended the RT-RAA detection methodology be used in a clinical test. For example, Zheng et al. developed the RT-RAA lateral flow dipstick assay based on the nucleocapsid (N) gene of SARS-CoV-2 in late 2019 (Zheng et al., 2021). In 2021, Li et al. established the RT-RAA method based on the highly conserved gene of epidemic and variant SARS-CoV-2 (Li et al., 2021). We previously developed a real-time RT-RAA detection approach for the coronavirus family swine epidemic diarrhea virus (PEDV) (Wu et al., 2022). To build a speedy, specific, simple, and low-cost methodology for the detection of the ALV, we constructed real-time RT-RAA primers and probes in the conserved region of the ALV P12 gene (Wang et al., 2020).

## Materials and methods

### Virus and cell culture

Harbin Pharmaceutical Group Bio-Vaccine Co., Ltd provided the Marek's disease virus (MDV, GenBank: L37202) vaccine (Harbin, China). Wens Foodstuff Group Co., Ltd supplied the avian reticuloendotheliosis virus (REV, not uploaded) (Yunfu, China). Newcastle disease virus (NDV, GenBank: JF950510), infectious bronchitis virus (IBV, GenBank: KR605489), infectious bursal disease virus (IBDV, GenBank: AF416621), infectious laryngotracheitis virus (ILTV, GenBank: JX458823), subgroup A avian leukemia virus (ALV-A, GenBank: DQ365814), subgroup J avian leukemia virus (ALV-J, GenBank: MT175600), subgroup K avian leukemia virus (ALV-K, GenBank: KP686144), subgroup H9N2 avian influenza virus (H9N2, GenBank: MN064851), and chicken infectious anemia virus (CIAV, GenBank: JX260426) were stored in our laboratory. The P12 gene of subgroup B avian leukemia virus (ALV-B, GenBank HM446005), subgroup C avian leukemia virus (ALV-C, GenBank J02342), subgroup D avian leukemia virus (ALV-D, GenBank D10652), and subgroup E avian leukemia virus (ALV-E, GenBank AY013303) were synthesized by Sangon Biotech (Shanghai) Co., Ltd. (Shanghai, China).

Cell culture DF-1 cells were purchased from the American Type Culture Collection. The cells were cultured in Dulbecco's modified Eagle medium (DMEM, Gibco; Thermo Fisher Scientific, Inc.) containing 10% fetal bovine serum (FBS, Gibco; Thermo Fisher Scientific, Inc.) and 1% penicillin/streptomycin (Gibco; Thermo Fisher Scientific, Inc.) at 37°C with 5% CO_2_ in a humidified incubator (Thermo Fisher Scientific, Inc.). The cells were inoculated with 44 clinical plasma samples of suspected ALV infection provided by Guangdong Ihealth Biotechnology Co., Ltd (Qingyuan, China) after they grew over 80%. After 7 days, we collected culture supernatant fluid.

### Reagents and instruments

Guangzhou Suyuan Biotechnology Co., Ltd. (Guangzhou, China) provided the AxyPrep Body Fluid Viral DNA/RNA Miniprep Kit. The RT-fluorescence nucleic acid amplification reagent (RAA method) was purchased from Nanning Zhuangbo Bio & Tech Co., Ltd. (Nanning, China). Guangzhou Qiyun Bio & Tech Co., Ltd. provided the pMD^TM^19-T Vector Cloning Kit (TAKARA, Guangzhou, China). In this investigation, a real-time fluorescence quantitative PCR apparatus (model: CFX96, Bio-Rad Laboratories, Shanghai, China) was employed.

### Schematic diagram for real-time RAA

**Figure 1:**
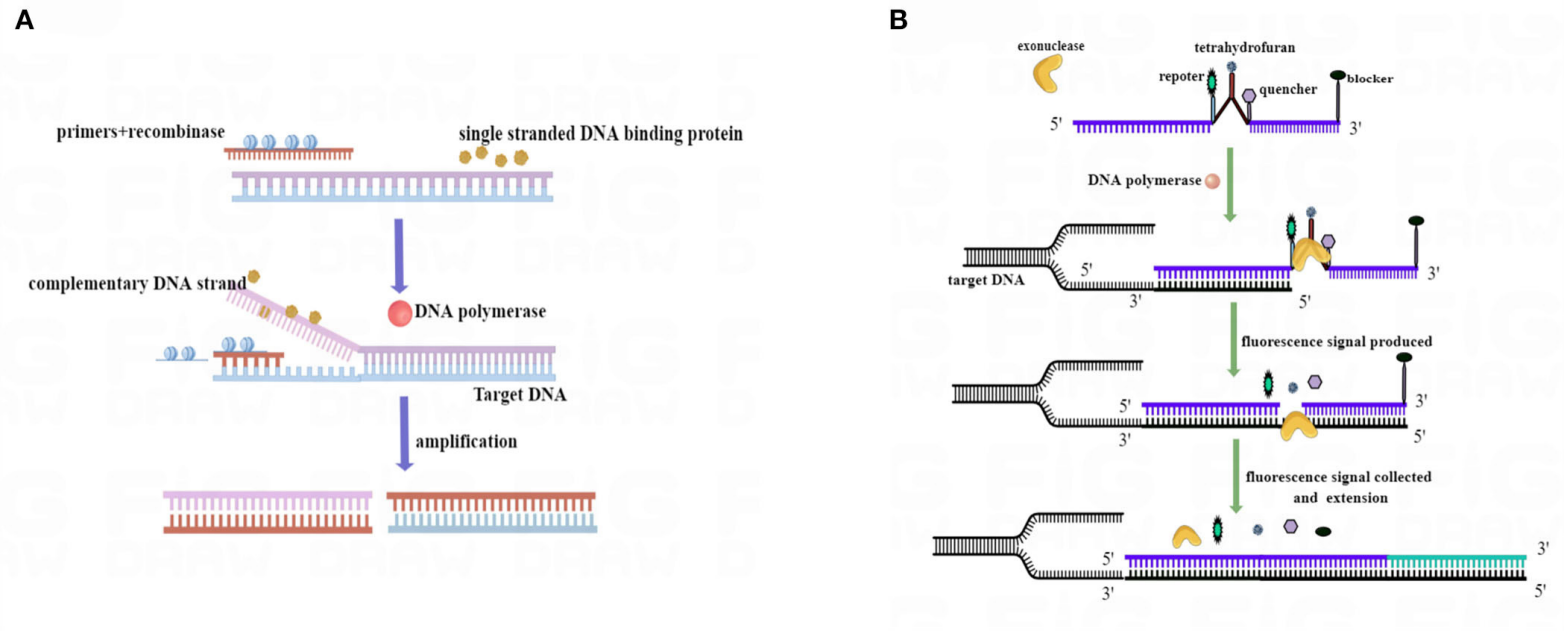
RAA process schematic diagram. **(A)** Mechanism based on the RAA process. **(B)** Mechanism of the real-time RAA probe amplification process. Exonuclease was activated when the probe and template DNA strands were bound. Exonuclease recognized tetrahydrofuran (THF) and cut at the site, separating reporter from quenched groups and generating a fluorescence signal to be collected. After the blocker separation, the probe was further extended in the template to complete the amplification. We drew this schematic diagram by Figdraw (www.figdraw.com).

Recombinase was bound to primers to form complexes, and the complexes searched for a specific binding site on the target DNA at a constant temperature. By utilizing single-stranded binding proteins, the double strand of template DNA is opened, and a single-stranded molecule is formed. In the next step, DNA polymerase was initiated at the 3′ end of the primer and synthesized toward the 5′ end of the target DNA. Hence, two new double-stranded DNA molecules were formed, and the cycle was repeated to achieve amplification (Chen et al., 2021; Wang et al., 2021) (Figure 1A). When nucleic acid exonuclease is not activated, the probe is stable and does not emit fluorescence signals; however, when the probe binds to the target DNA site, exonuclease was activated. Upon recognizing tetrahydrofuran (THF) on the probe, nucleic acid exonuclease cleaves both the reporter group and the burst group, and the reporter group is released, allowing the fluorescence signal to be emitted. As the probe was amplified further to the 3′ end, the blocker was cut off, and the probe continued to amplify to the 5′ end of the target DNA. Consequently, real-time fluorescence signals were gathered and plotted (Figure 1B).

### Real-time RT-RAA primers and probe design

We downloaded the sequences of the ALV from the GenBank database (GenBank accession numbers: Z46390, MT175600, MF817820, AF052428, HM446005, JX855935, KY581580, MG770235, KP686142, KY851580, MG770235, MT319751, DQ365814, HM452340, KU375453, KY612442, MF926337, AF052428, HM446005, KM873223, and D10652) and used DNAMAN (Lynnon Biosoft, version: v9.0., California, USA) and DNASTAR (DNASTAR, Inc., Madison, USA) to analyze and align multiple sequences. Based on the sequence alignment results, we designed primers and probes (Table 1) for real-time fluorescence RT-RAA on ALV HPRS103 (GenBank accession number: Z46390) of the P12 gene. PCR primers (Table 1) of ALV LTR, previously reported by Yan (2020) for clinical sample detection, are used in this study. The primers and probes utilized in this examination were synthesized by Sangon Biotech (Shanghai) Co., Ltd. (Shanghai, China).

**Table 1:** Primers and probe sequences used in the study.

**Primer**	**Sequence (5′-3′)**	**Localization**	**Size of product (bp)**
RAA-F	TTATCAGGCGCAGTGCCCGAAAAAACG	2,154–2,180	114
RAA-R	CCCTGGTTGCCATCCCGCTTCCTACACTGTC	2,240–2,270	
RAA-P	AATCAGGAAACAGCCGTGAGCGATGTCAGT/i6FAMdT//THF//iBHQ1dT/ GTGACGGGATGGGAC[C3-spacer]	2,183–2,230	
P12-F	TCGTCTGCTATCCAGCCCTTAG	2,041–2,062	323
P12-R	CGATCTCTATGTTCCATTGTCA	2,342–2,363	
LTR-F	TGCCTGTAGTGATTAAGACA	1,319–1,338	673
LTR-R	TCTAGCACATATTTGATTAT	1,972–1,991	

### Generation of plasmid standard

Targeted P12 (ALV-J, GenBank accession number MT175600: 2041-2363) gene fragments of ALV were amplified by the PCR primers and are shown in Table 1, and the amplified product was then cloned into pMD^®^19-T vectors (TaKaRa, China). After extraction, the concentration of this plasmid was evaluated, and the copy number of the ALV P12 gene was calculated to be around 2.41 × 10^10^ copies/μl.

### Sample preparation

We extracted MDV, CAV, IBV, NDV, IBDV, H9N2, ILTV, REV, and the 44 culture supernatant fluid samples using the AxyPrep Body Fluid Viral DNA/RNA Miniprep Kit (Guangzhou, China). The nucleic acids were then stored in the ultralow-temperature freezer at −80°C in our laboratory.

### Verification of RT-RAA primers

In testing the size of the real-time RAA primers, the positive control was ALV nucleic acid, and the negative control was ddH_2_O. The RT-PCR system required RAA-F (10 μM) 1.0 μl, RAA-R (10 μM) 1.0 μl, RNase-free ddH_2_O 5.0 μl, One Step Enzyme Mix 1 μl, 2 One Step Mix 10.0 μl, and RNA 2 μl. The RT-PCR was performed at 50°C for 30 min, 94°C for 3 min, 94°C for 10 s, 58°C for 30 s, 35 cycles, and 72°C for 5 min. On a 1.5 percent agarose gel, the RT-PCR products were analyzed.

### The system of real-time RT-RAA reaction

The real-time RT-RAA reaction system is made up of the following components: RAA-F (10 μM) 2.0 μl, RAA-R (10 μM) 2.0 μl, RAA-P (10 μM), 2.0 μl, RT-RAA reaction unit 1 tube, A buffer 25 μl, and B buffer 2.5 μl. The total volume was 50 μl, with 12.9–15.9 μl of ddH_2_O and 2–5 μl of templates. The fluorescence signals were acquired after one cycle of 39°C for 60 s and 40 cycles of 39°C for 30 s of real-time RT-RAA. After the reaction, the positive control had a smooth amplification curve, and the Ct value was ≤39; the negative control had no amplification curve, or the Ct value was >39, showing the assay had valid results.

### Assay for RT-RAA sensitivity

Serial dilutions of the standard plasmid of ALV P12 were carried out 10 times by ddH_2_O. We employed blood nucleic acid of SPF chickens as the dilution solution of the standard plasmid (complex plasmid solutions) of ALV P12 to more effectively apply to clinical sample detection. A total of six concentration gradients served as templates, which were 10^5^ copies/μl, 10^4^ copies/μl, 10^3^ copies/μl, 10^2^ copies/μl, 10^1^ copies/μl, and 10^0^ copies/μl. The sensitivity assays of two different dilution methods were then repeated three times.

### Assay for the RT-RAA specificity

The nucleic acid of ALV-A, ALV-B, ALV-C, ALV-D, ALV-E, ALV-J, and ALV-K was a positive control and ddH2O was a negative control. For specificity analysis of MDV, CAV, IBV, NDV, IBDV, H9N2, ILTV, and REV nucleic acids, 2 μl of templates were obtained and repeated three times.

### Assay for the RT-RAA repeatability

The complex solution plasmids and standard plasmids dilution gradients were 10^3^ copies/μl, 10^2^ copies/μl, and 10^1^ copies/μl were taken at 5 μl, and repeating assays were three times respectively.

### Analysis of the clinical sample test results

Nucleic acids from the 44 culture supernatant fluid samples suspected of being infected with ALV were used to detect by RT-PCR and real-time RT-RAA. We compared and analyzed the results of RT-PCR and real-time RT-RAA to evaluate the method we established in this study.

## Results

### RT-RAA primer verification

**Figure 2:**
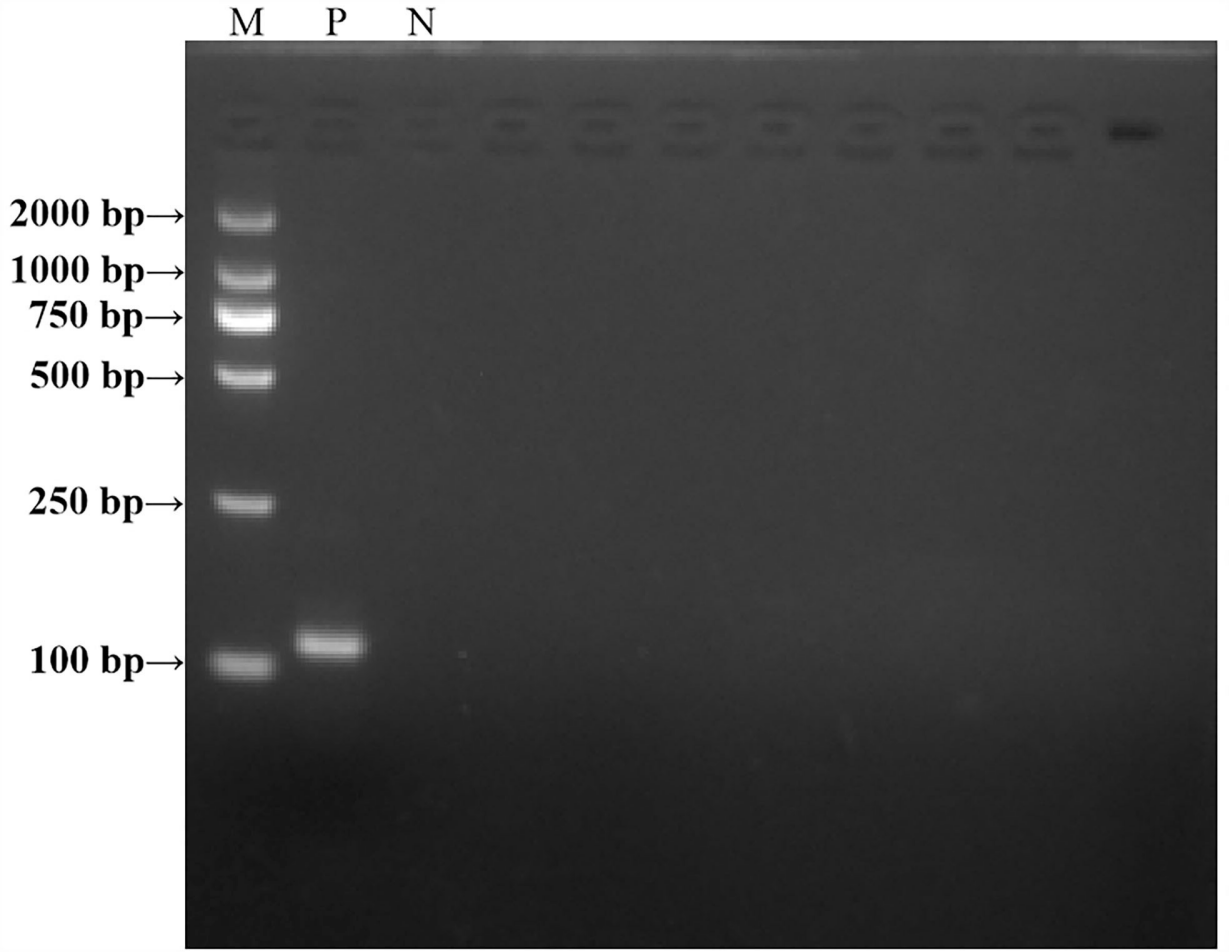
Agarose electrophoresis PCR-amplified products verified the results of the RT-RAA primer size. M, D 2000 Marker; P, positive control, that is, ALV nucleic acid; N, negative control, that is, ddH_2_O.

A PCR experiment was conducted to verify the size of the real-time RAA primers. The results are shown in Figure 2. The positive control real-time RAA primer product was a particular band and no-primer-dimers with 114 bp, which we expected. The negative control was nonspecific bands and no-primer-dimers. As a result, the real-time RT-RAA primers were able to detect ALV precisely.

### Assay for the RT-RAA sensitivity

**Figure 3:**
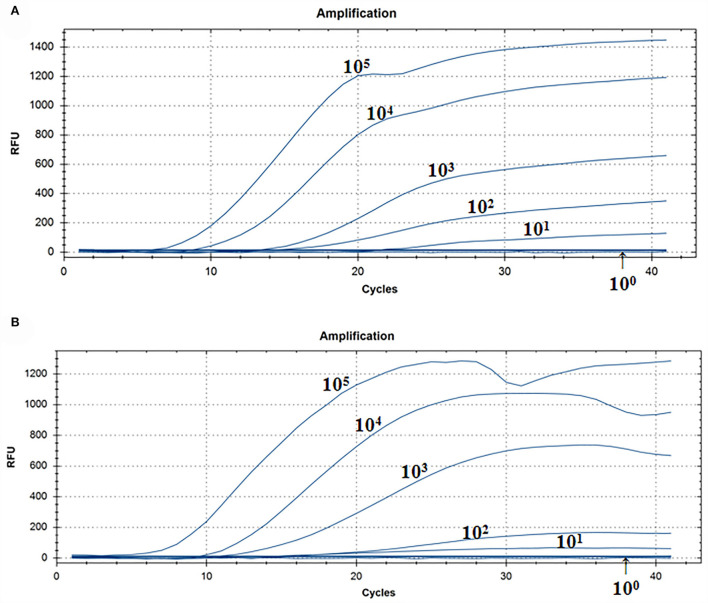
Assay for the real-time RT-RAA sensitivity. **(A)** Pure standard plasmids were detected at 10^0^, 10^1^, 10^2^, 10^3^,10^4^, and 10^5^ copies/μl, respectively. **(B)** Complex plasmid solutions were detected at 10^0^, 10^1^, 10^2^, 10^3^,10^4^, and 10^5^ copies/μl, respectively. The real-time RAA can detect 10 copies per reaction in pure standard plasmids and complex plasmid solutions.

A representative real-time RT-RAA result was chosen from the three independent assays, as shown in Figure 3. The result of standard plasmids of ALV P12 showed that the detection limit of each reaction was about 10^1^ copies/μl (Figure 3A). The complex plasmid solutions of ALV P12 also showed that the detection limit of each reaction was about 10^1^ copies/μl (Figure 3B). Based on these results, we can know that the detection limit of real-time RAA was 10 copies per reaction.

### Assay for the RT-RAA specificity

**Figure 4:**
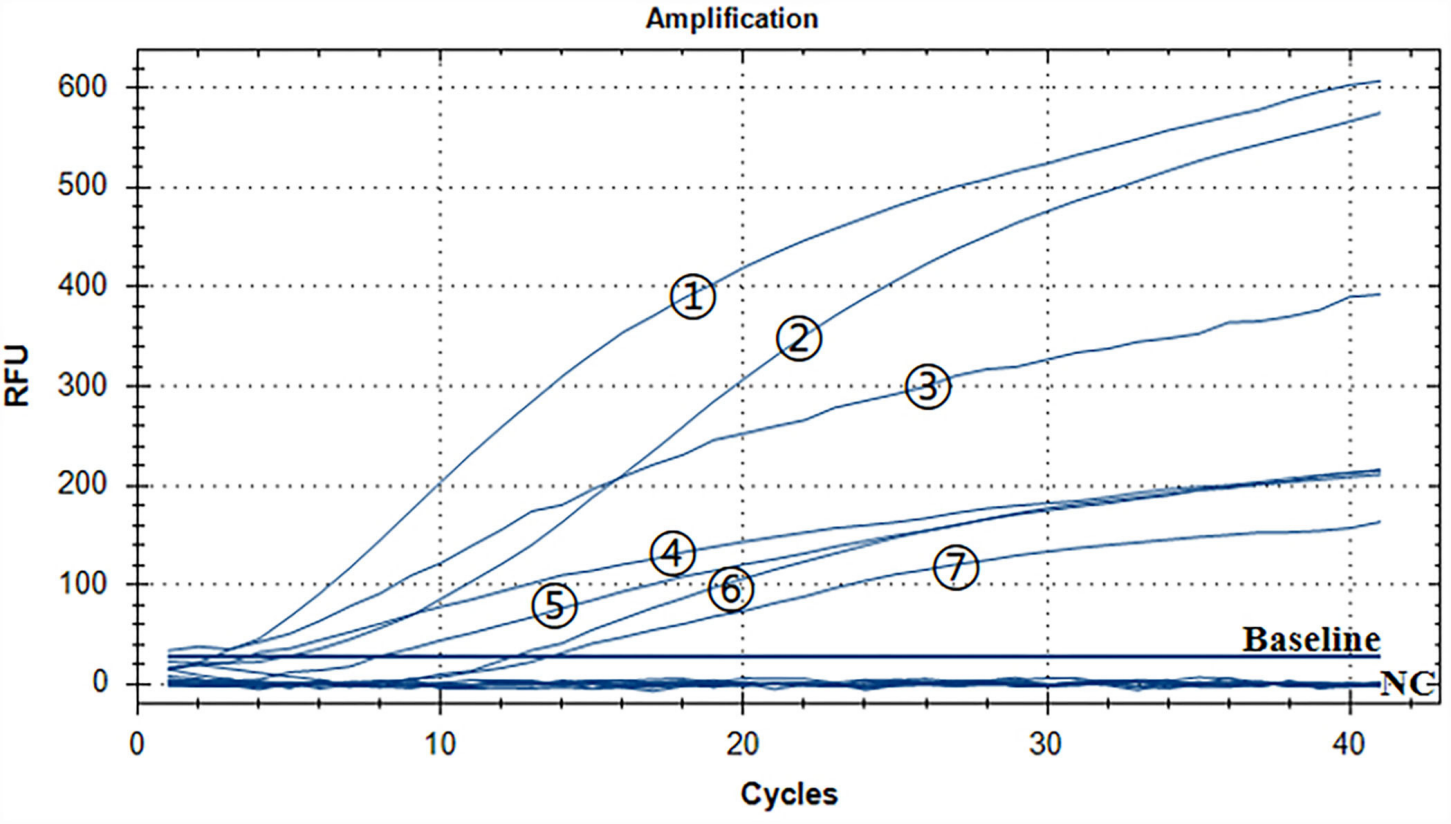
Assay for the RT-RAA specificity. The positive control was ALV-A, B, C, D, E, J, and K nucleic acids, and the negative control was MDV, CAV, IBV, NDV, IBDV, H9N2, ILTV, and REV nucleic acids and ddH_2_O. - respectively: ALV-J, ALV-K, ALV-A, ALV-D, ALV-E, ALV-C, and ALV-B.

A representative real-time RAA result was chosen from the three independent assays, as shown in Figure 4. The groups with ALV-A, B, C, D, E, J, and K nucleic acids were positive controls, and both of them had fluorescence signals. The negative control including ddH_2_O, MDV, CAV, IBV, NDV, IBDV, H9N2, ILTV, and REV nucleic acid had no fluorescence signal. Therefore, we developed a real-time RT-RAA approach for detecting the ALV that had no cross-reactivity with MDV, CAV, IBV, NDV, IBDV, H9N2, ILTV, or REV.

### Assay for the RT-RAA repeatability

**Figure 5:**
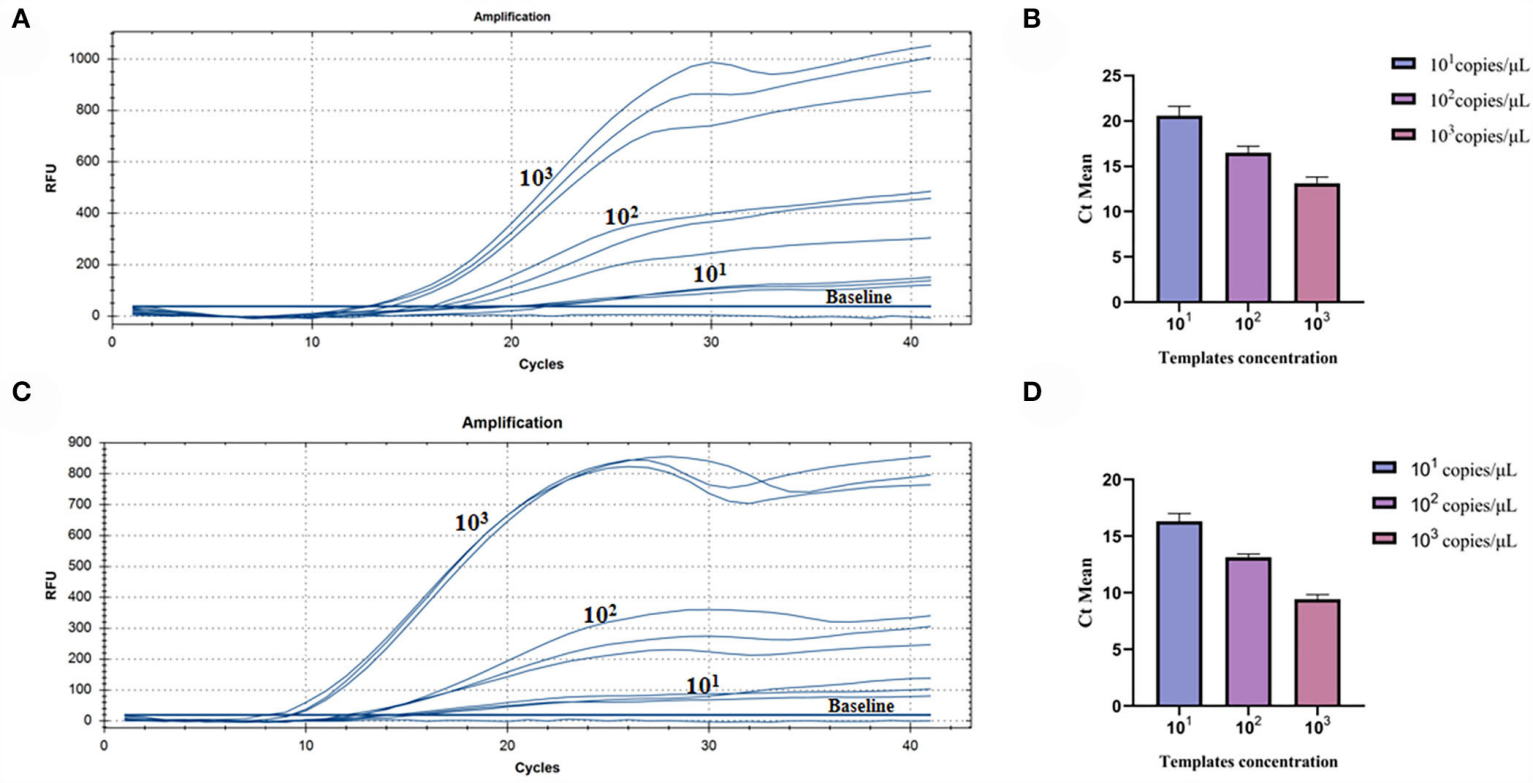
Assay for the RT-RAA repeatability: **(A)** Three low-concentration gradients of the pure plasmid standards were measured, each repeated three times: 10^1^, 10^2^, and 10^3^ copies/μl. **(B)** Data from three runs of pure plasmid standards to analyze real-time RAA assay. **(C)** Three low-concentration gradients of the complex plasmid solutions were measured, each repeated three times: 10^1^, 10^2^, and 10^3^ copies/μl. **(D)** Data from three runs of complex plasmid solutions to analyze real-time RAA assay.

The repeatability of inter-assay detection was estimated using the plasmid standards of three low-concentration gradients as templates. The results of pure standard plasmids are shown in Table 2 and Figures 5A,B, where the coefficients of variation (CV) were 5.11%, 4.28%, and 5.18%. The results of complex plasmid solutions are shown in Table 3 and Figures 5C,D, where the coefficients of variation (CV) were 4.33%, 2.38%, and 4.43%. In the two different dilution methods, the ultrafine template concentration of the 10^1^ copies/μl was the same in three repetitions, denoting good repeatability.

**Table 2:** Real-time RT-RAA repeatability assay of pure plasmid standards.

**Pure plasmid standards** **(copies/μl)**	**Ct (Mean ± SD)**	**CV%**
10^1^	20.57 ± 1.05	5.11
10^2^	16.51 ± 0.71	4.28
10^3^	13.14 ± 0.68	5.18

**Table 3:** Real-time RT-RAA repeatability assay of complex plasmid solutions.

**Complex plasmid** **solutions** **(copies/μl)**	**Ct (Mean ± SD)**	**CV%**
10^1^	16.30 ± 0.71	4.33
10^2^	13.12 ± 0.31	2.38
10^3^	9.43 ± 0.42	4.43

### Analysis of the clinical sample test results

As shown in Table 4, of all 44 clinical samples of nucleic acids, RT-PCR, and real-time RT-RAA assays revealed positive amplification of 24 nucleic acids. However, two of the nucleic acids were positive amplification by real-time RT-RAA assay, and it was negative amplification by RT-PCR assay. RT-PCR and real-time RT-RAA had a sensitivity of 92.31% and a specificity of 100%. The kappa value of this assay was 0.91 (>0.81) when compared to the RT-PCR assay, indicating that the correlation between the two assays was close to perfect.

**Table 4:** Performance of the real-time RT-RAA assay compared to the reference method, RT-PCR, for detecting ALV in clinical samples.

**Assay**		**RT-PCR**	**real-time RT-RAA**
Positive		24/44	26/44
Negative		20/44	18/44
Performance	Sensitivity (%)	92.31	
characteristics	Specificity (%)	100	
	Kappa	0.91	

A total of 44 clinical samples of suspected ALV infection were provided by Guangdong Ihealth Biotechnology Co., Ltd (Qingyuan, China). The qualitative detection of the real-time RAA on clinical samples was conducted by using the positive control had a smooth amplification curve, and the Ct value was ≤ 39, the negative control has no amplification curve, or the Ct value was >39, showing the assay has valid results.

## Discussion

Currently, the gold standard for ALV diagnostic tests was virus isolation, which was combined with ELISA to produce results (Zhou et al., 2019). However, this method was hampered by the professional requirements of testing personnel, laboratory environment, long turnaround time (over 7 days), high cost, and large reagent consumption. As to the complex epidemic of ALV, researchers have established many detection methods in different directions, such as virus isolation, reverse transcription–polymerase chain reaction (RT-PCR) (Garcia et al., 2003; Gao et al., 2014), quantitative reverse transcription–polymerase chain reaction (RT-qPCR) (Dai et al., 2015; Chen et al., 2018), reverse transcription–loop-mediated isothermal amplification (RT-LAMP) (Zhang et al., 2010; Peng et al., 2015) of molecular diagnostic, ELISA (De Boer and Osterhaus, 1985; Yun et al., 2013; Chang et al., 2020), and indirect immunofluorescence assays (IFA) (Pham et al., 1999; Zhang et al., 2021) of serological diagnostic. However, these methods have some weak points, for instance, RT-PCR and RT-qPCR require expensive and cumbersome instruments, and 2–3 pairs of primers for RT-LAMP tend to cause false positives because of nonspecific binding. Combined with the aforementioned detection methods, the prevention and control of ALV are difficult because it has limited clinical tests and cost the laboratory labor force and resources. To improve this situation, we verified the RT-RAA detection method through a series of experiments and validated the real-time RT-RAA technology for the detection of ALV in clinical samples compared with RT-PCR. This method is a breakthrough as it requires less time and does not require expensive and bulky equipment to regulate temperature. It also enables detection in remote breeding sites and maintains the same sensitivity and accuracy as RT-qPCR (Chen et al., 2018).

Over time, the genome of ALV is constantly mutating and recombining, mainly focusing on the gp85 gene (Gao et al., 2012; Jiang et al., 2014; Wang et al., 2017), and new subgroups are likely to emerge soon. According to the molecular epidemiological investigation of ALV in China for many years, the P12 gene was highly conservative (Lin et al., 2017; Wang et al., 2020). Therefore, we selected this region to design an amplification fragment of about 114 bp for the real-time RT-RAA primers and probe, ensuring that subgroups of ALV could be detected, including new subgroups that may appear in future.

The detection time of real-time RT-RAA was as fast as 5 min, and within 30 min the assay was completed, which could specifically detect ALV-A, B, C, D, E, J, and K, and without cross-reaction with MDV, CAV, IBV, NDV, IBDV, H9N2, ILTV, and REV. Because this method can be combined with a portable GENCHEK fluorescence detector, it improves analysis reliability in complex and poor environments. The reported assay reliably has a limit of detection of approximately 10 copies of the virus per reaction both in pure standard plasmids and complex plasmid solutions, with dilutions ranging from approximately 10^1^ to 10^5^ expected copies. Notably, the accuracy of the validated assay was consistent with that of the clinically validated RT-PCR assay for 44 ALV plasma samples therein, performance characteristics of sensitivity were 92.31%, specificity was 100%, and data analysis kappa = 0.91 (kappa > 0.81).

In reality, most breeding industries choose ELISA when performing NEP because it does not need a specific environment and complex operation. However, the accuracy of the result needs to be improved (Tsukamoto et al., 1991; Zhou et al., 2019). While real-time RT-RAA has the advantages of ELISA, it also can maintain accurate detection results within the lowest sensitivity, with a CV of <5.18% in pure standard plasmids and 4.43% in complex plasmid solutions. We recommend that it could be used as the preferred method for NEP of ALV. There have been some studies developed with qPCR for the general detection of ALV, such as Qin et al. for ALV-J, Chen et al. for ALV-K, and Dai et al. for ALV-A and ALV-B that have developed multiplex qPCR (Qin et al., 2013; Dai et al., 2015; Chen et al., 2018). As we mentioned earlier, the ALV has 11 subgroups, and the poultry industry monitoring of the main epidemic exogenous subgroups by qPCR is also beneficial to the prevention and control of the ALV. However, these methods often ignore the detection of endogenous ALV-E, whereas real-time RAA can be applied to the whole subgroups of ALV.

These preliminary results show that RT-RAA is a promising general-purpose technology, which requires no professional personnel and is adaptive to different environments. It not only enables laboratory clinical diagnosis to produce accurate results more quickly and saves reagent costs but also overcomes the bottleneck of current assay methods for ALV. Real-time RT-RAA will be well applied to the large-scale industry and the remote retail farmers by performing NEP in ALV; meanwhile, it can be suitable for the new subgroup of ALV in future.

## Conclusion

This study successfully developed a real-time RT-RAA method to detect ALV within 30 min at a constant temperature. The real-time RT-RAA method has the advantages of high sensitivity, strong specificity, good repeatability, and low cost, which could be key factors in NEP ALV in the vast poultry industry in China.

## Data availability statement

The raw data supporting the conclusions of this article will be made available by the authors, without undue reservation.

## Ethics statement

Institutional and national guidelines for the use and care of laboratory animals were strictly followed. The use of animals in this study was approved by the South China Agricultural University Committee for Animal Experiments (approval ID: SYXK2019-0136).

## Author contributions

XW conducted the experiments, analyzed the data, and wrote the manuscript. FChu, SC, HZ, and HH conducted the experiments. LZ, LG, JW, and MC analyzed part of the data. FChe and ZX checked and finalized the manuscript. QX and XW designed the experiments, modified the manuscript, and supervised the whole work. All authors read and approved the final manuscript.

## Funding

This work was funded by the Key Research and Development Program of Guangdong Province (grant number 2020B020222001), the National Natural Science Foundation of China (grant numbers 31902252 and 31972659), Guangdong Basic Research Foundation (grant numbers 2019B1515210034, 2019A1515012006, 2021A1515010817, and 2022A1515012115), the Chief Expert Project of Agricultural Industry Technology System in Guangdong Province (grant numbers 2019KJ128 and 2020LJ128), and the Special Project of National Modern Agricultural Industrial Technology System (grant number CARS-41).

## Conflict of interest

The authors declare that the research was conducted in the absence of any commercial or financial relationships that could be construed as a potential conflict of interest.

## Publisher's note

All claims expressed in this article are solely those of the authors and do not necessarily represent those of their affiliated organizations, or those of the publisher, the editors and the reviewers. Any product that may be evaluated in this article, or claim that may be made by its manufacturer, is not guaranteed or endorsed by the publisher.
